# Resting-state EEG microstate features for Alzheimer’s disease classification

**DOI:** 10.1371/journal.pone.0311958

**Published:** 2024-12-12

**Authors:** Xiaoli Yang, Zhipeng Fan, Zhenwei Li, Jiayi Zhou

**Affiliations:** School of Medical Technology and Engineering, Henan University of Science and Technology, Luoyang, China; University of Bucharest, Faculty of Biology, ROMANIA

## Abstract

Resting-state electroencephalogram (EEG) microstate analysis resolves EEG signals into topographical maps representing discrete, sequential network activations. These maps can be used to identify patterns in EEGs that may be indicative of underlying neurological conditions. One such pattern is observed in EEGs of patients with Alzheimer’s disease (AD), where a global microstate disorganization is evident. We initially investigated the classification efficacy of microstate parameters as markers for AD classification. Subsequently, we compared the classification efficacy of EEG conventional features to ascertain the superiority of microstate features. We extracted raw EEG data from a public, independent database, OpenNeuro EEG. The raw EEG was subjected to preprocessing and band-pass filtering to obtain five distinct frequency bands. The SVM classifier was used to input the microstate feature set to determine the one with the best classification effect as the main band. In order to verify the advantage of the microstate features, the AD group and the healthy control group were filtered for the main frequency bands respectively. Then the microstate feature set and the regular feature set were extracted. The two feature sets were input into four different conventional machine learning classifiers, namely SVM, KNN, RF, and LR, in order to avoid the classifiers as the dependent variable. And the comparison of the classification results of simply two feature sets as the dependent variable can be obtained. The results show that in the Alpha (8–13 Hz) sub-band, the microstate feature set as model input to SVM is optimal for the recognition of AD, with a classification accuracy of 99.22%. The Alpha band, as the main frequency band, the microstate feature set as model input to the four classifiers obtains an average classification accuracy of 98.61%, and the average classification accuracy obtained by the conventional EEG feature set as model is 91.19%. Based on four different classifiers, microstate parameters can be served as markers to effectively classify the EEG of AD patients. The microstate feature set outperforms the conventional EEG feature set after excluding the effect of classifiers.

## Introduction

The global improvement of health security services is contributing to an increase in human life expectancy across the majority of the world’s regions. As healthcare standards rise, it is anticipated that life expectancy in developing countries will also grow [1]. However, an increase in life expectancy also results in a greater likelihood of developing age-related brain diseases, which can have a profound impact on our daily lives and even lead directly to death. The human brain is one of the most complex organs in humans and is also the least explored part of the human body. The reasons behind many functional disorders of the brain remain poorly understood [2]. AD is a complex neurological disease that has a significant impact on patients’ financial and medical well-being. It is considered by research to be the leading cause of dementia and is characterized by dementia, neuronal loss, and brain atrophy. According to the survey, more than 9.9 million new cases of AD are diagnosed annually worldwide. It is projected that the number of AD patients will be approximately 82 million by 2030 and 152 million by 2050 [3]. Furthermore, the etiology of AD remains uncertain, with approximately 70% of the risk attributed to genetic factors. Despite decades of research, there is currently no medication that can halt the progression of AD at its source. Current therapeutic approaches only provide symptomatic relief and do not offer a definitive cure or protection. The diagnosis of Alzheimer’s disease is now commonly performed through screening procedures that include an analysis of clinical signs, an evaluation of health information, family counseling, and clinical, neurological, and psychiatric examinations. Additionally, neuropsychological testing can be employed as a tool for detecting objective indications of memory disorders at an early stage. In clinical practice, the early diagnosis of AD is of paramount importance. EEG has a high temporal resolution [4], which allows for the objective extraction of certain parameters that are widely used in the diagnosis of AD. However, obtaining EEG data from patients with mild cognitive impairment (MCI) or AD is a challenging process. In contrast to ECG and other biomedical records, these databases are not accessible to the general public. Consequently, it is challenging to conduct continuous benchmarking and evaluation of the most advanced methods for detecting AD from EEG signals [5]. However, in comparison to other methods of acquiring brain information from AD patients, the cost of obtaining EEG signal data is relatively low, and patient cooperation during the acquisition process is high.

The performance of EEG allows for the measurement of brain electrical activity on a millisecond scale, which is commensurate with the neuronal firing rate. Consequently, if some objective features of the EEG could be used for early diagnosis of AD, it would greatly reduce the stress on physicians and patients. Furthermore, given that distinct EEG topographic representations are responsive to varying functional states, analysis of spontaneous EEG over brief time periods has revealed the presence of multiple topographic directions that undergo rapid transitions [6]. Resting-state EEG is a functional imaging modality that has been extensively utilized to delineate alterations in brain function in individuals diagnosed with AD [7].

It has been posited that the EEG of AD patients exhibits reduced high-frequency power, increased low-frequency power, and impaired interhemispheric coherence when compared to controls [8, 9]. In 1987, Lehmann et al. observed that the scalp potential topography remained unchanged for a relatively short period of time, typically between 80 and 120 milliseconds. Thereafter, it rapidly switched to the next scalp potential topography [10]. The investigation revealed that the number of these inter-switching scalp potential topographies was limited, and that these microstate categories were repeatable and observable across treatments and subjects [11]. Spatial clustering ultimately leads to four classes of classical microstates that explain 80% of the total variation in resting EEG [12]. The duration, coverage, field power, and transfer probability of microstates in the EEG of patients with AD may be influenced by underlying brain disease processes. The onset and duration of microstate topology were not consistent between AD patients and healthy controls. Therefore, it can be demonstrated that patients with AD exhibit a more pronounced manifestation of anterior and posterior microstate topologies [13–15].

In 1993, Ihl and colleagues observed that patients with AD exhibited longer overall microstate durations compared to healthy older adults [16]. Although the results were derived from smaller clinical studies, they demonstrated a relationship between cognitive state and microstate parameters. Subsequently, Dierks T. (1997) found shorter microstate durations in patients with different stages of cognitive impairment and AD by dividing the EEG in the time domain into segments with similar spatial distribution on the scalp (microstates) [17]. Furthermore, the study indicated that the stability of EEG microstates is diminished in patients with Alzheimer’s disease. Koenig et al. (2005) also reached similar conclusions with synchronized global time and frequency domain measurements [18]. Smailovic et al. (2019) demonstrated that microstates may serve as new functional state and feature markers of synchronized brain activity. This contributes to the understanding and detection of early disruptions of the neurocognitive network in patients on the AD continuum. Tait et al. (2020) observed an increase in the average duration of microstates in patients with AD [19]. A review of the literature reveals inconsistencies in reports of altered microstate duration. A comparison of the data revealed that there were differences in microstate duration when the EEG was recorded with the eyes open versus the eyes closed. In their 2022 study, Shi and colleagues employed microstate analysis to assess the severity of patients with AD and MCI and to effectively differentiate between AD and MCI. The findings revealed that there were discrepancies in microstate temporal profiles between the groups, suggesting that microstates can serve as neurobiological markers for the categorization of AD [20]. Yan et al. (2024) observed that patients with AD exhibited longer durations of microstates C and D and a reduced occurrence of microstate B compared to healthy controls. The authors employed EEG microstates as a means of predicting AD classification, achieving a moderate level of accuracy [21]. It is proposed that alterations in EEG microstate patterns in patients with AD are associated with cognitive function and disease severity.

Previous studies have employed microstates for early diagnosis, yet without sufficient methodological and variable controls. In this study, we utilized multiple machine learning algorithms for comparison and combined them with microstate feature parameters. The objective was to identify the machine learning algorithms most suitable for classifying AD with healthy controls. Furthermore, this study will ascertain whether machine learning classification of the microstate parameter feature set is more effective than the conventional EEG feature set.

## Materials and methods

### Dataset

A publicly accessible dataset was available for this study [22]. The dataset contains EEG resting state eye closure recordings from 58 subjects. The data were acquired using 21 leads. There were 29 cases of Alzheimer’s disease (AD group) and 29 healthy individuals (HC group). The cognitive and neuropsychological status of the subjects was evaluated using the International Mini-Mental State Examination (MMSE) as illustrated in Table 1. The MMSE scores ranged from 0 to 30, with lower scores indicating a more severe cognitive decline. With regard to the AD group, the presence of comorbidities associated with dementia was excluded. Similarly, regarding the AD group, the presence of dementia-related comorbidities was excluded. The mean Mini-Mental State Examination (MMSE) score was 17.75 (standard deviation [SD] = 4.5) in the Alzheimer’s disease (AD) group and 30 in the healthy control (HC) group. The mean age was 66.4 years (SD = 7.9) in the AD group and 67.9 years (SD = 5.4) in the HC group. The authors confirm that informed consent was obtained from all subjects for the collection of this dataset. In accordance with the stipulations of the General Data Protection Regulation (GDPR), the participants have been anonymized, and their personal information has been withheld from disclosure.

**Table 1:** Gender, age, group affiliation and MMSE scores of the subjects.

Participant_ID	Gender	Age	Group	MMSE
001	F	57	A	16
002	F	78	A	22
003	M	70	A	14
004	F	67	A	20
005	M	70	A	22
006	F	61	A	14
007	F	79	A	20
008	M	62	A	16
009	F	77	A	23
010	M	69	A	20
011	M	71	A	22
012	M	63	A	18
013	F	64	A	20
014	M	77	A	14
015	M	61	A	18
016	F	68	A	14
017	F	61	A	6
018	F	73	A	23
019	F	62	A	14
020	M	71	A	4
021	M	71	A	22
022	F	68	A	20
023	M	60	A	16
024	F	69	A	20
025	F	79	A	20
026	F	61	A	18
027	F	67	A	16
028	M	49	A	20
029	F	53	A	16
030	F	56	A	20
031	F	67	A	22
032	F	59	A	20
033	F	72	A	20
034	F	75	A	18
035	F	57	A	22
036	F	58	A	9
037	M	57	C	30
038	M	62	C	30
039	M	70	C	30
040	M	61	C	30
041	F	77	C	30
042	M	74	C	30
043	M	72	C	30
044	F	64	C	30
045	F	70	C	30
046	M	63	C	30
047	F	70	C	30
048	M	65	C	30
049	F	62	C	30
050	M	68	C	30
051	F	75	C	30
052	F	73	C	30
053	M	70	C	30
054	M	78	C	30
055	M	67	C	30
056	F	64	C	30
057	M	64	C	30
058	M	62	C	30

### EEG data preprocessing

In this paper, we utilized the MNE tool library for Python to preprocess the EEG dataset with the objective of obtaining artifact-free EEG features for microstate analysis. EEG features are properties of data that can be extracted from EEG signals. Selecting the optimal features can assist in reducing processing costs and improving classification [23]. The more established previous preprocessing pipeline was followed. Four classical microstates are applied. The entire process of extracting EEG features from raw data is as follows [24]. The EEG was re-referenced to a common averaging electrode and filtered using an 8–13 Hz bandpass filter. The continuous EEG data was segmented 5-second non-overlapping elements, allowing for the removal of elements containing artifacts and the extraction of EEG features from the raw data. 700s of resting-state EEG was taken for each subject, and after segmentation we obtained 4060 artifact-free periods for Alzheimer’s disease patients and 4060 artifact-free periods for healthy subjects. This approach avoided selecting and analyzing only some of the artifact-free data points, but rather all phases were used for classification [25]. The final stage of preprocessing entails the extraction of microstate features and conventional EEG features for each calendar element of the AD group and the healthy control group, respectively. The minimum and maximum k-folds were applied to the dataset, but this resulted in a reduction in efficiency due to variations in the percentage of training and testing data. These values are selected when the dataset is insufficient and can be used as a general indicator of the model’s average performance. As a result of the augmentation of the dataset, the quantity of data obtained is more objective [26]. The dataset was thus divided into a training set comprising 70% of the data and a testing set comprising 30% of the data using the train_test_split() function. In turn, the microstate feature classification model and the conventional EEG feature classification model used for classification can be obtained.

### Microstate analysis

EEG microstates were first proposed by Lehmann et al. (1987). The researchers observed that the EEG was made up of several typical patterns that stayed the same for about 80 to 120 milliseconds before changing. These cycles of quasi-steady-state EEG topographies are referred to as microstates [27]. Microstate analysis comprises two principal stages. Initially, EEG data is segmented in order to identify the most representative template maps corresponding to different microstate categories. Subsequently, these categories are fitted back to the EEG data in order to obtain a microstate sequence [28].

For the analysis of microstates, the Pycrostates tool library for EEG analysis in Python was employed. Initially, the field strength (global field strength (GFP)) was calculated at each moment. The GFP was defined as:

$$\text{GFP}=\sqrt{\frac{1}{\text{N}}\sum_{\text{i}=1}^{\text{N}}{\left({\text{u}}_{\text{i}}-\bar{\text{u}}\right)}^{2}}$$

where N denotes the number of electrodes, u_i_ is the voltage of u at electrode i, and $\bar{\text{u}}$ is the average voltage of all electrodes on topographic map u. The GFP values are higher when there are significant positive and negative peaks in the scalp potential field, whereas a potential field with a flat gradient leads to lower GFP values. To achieve a smoother representation of the GFP, a Gaussian-weighted shift was applied at 50 time points. The EEG topographies at the peaks of the smoothed GFP were selected for further clustering. The data set was clustered and analyzed using the modified K-means algorithm to identify the template microstates. The K-means algorithm is a distance-based clustering algorithm that employs distance as the evaluation index of similarity. In this context, the degree of similarity between two objects is considered to be inversely proportional to the distance between them. One advantage of this approach is that it is well-suited to handling large-scale datasets. This is because the computational complexity of the method is primarily determined by the size of the data, rather than the volume of the data [29]. The optimal number of microstate classes is automatically determined based on the global explanatory variance (GEV) and global mapping dissimilarity (GMD). GEV quantifies the percentage of the data that can be explained by the microstate classes [30]. GMD is employed to quantify the topographic variation of the microstate classes. Cluster analysis was initially conducted at the individual level. Thereafter, the template map for each subject was subjected to a second clustering procedure, with the objective of identifying the most dominant cluster among all subjects [31].

In the quadratic fitting process, each time point of each calendar epoch was assigned to a specific microstate. Specifically, we computed the spatial correlation between the instantaneous EEG topography and each microstate category. Each sampling point was labeled according to the microstate with the highest correlation. To maintain the stability of the microstate labeling, temporal smoothing was employed to avoid the interference of noise. In essence, the stochastic K-means algorithm can be run multiple times to test multiple segmentations on the same dataset. The GEV criterion can then be employed to select the optimal stochastic K-means algorithm for further analysis. GEV is a measure of the degree of similarity between each EEG sample and the microstate prototypes to which it has been assigned. A higher GEV value indicates a more accurate segmentation [32].

**Fig 1:**
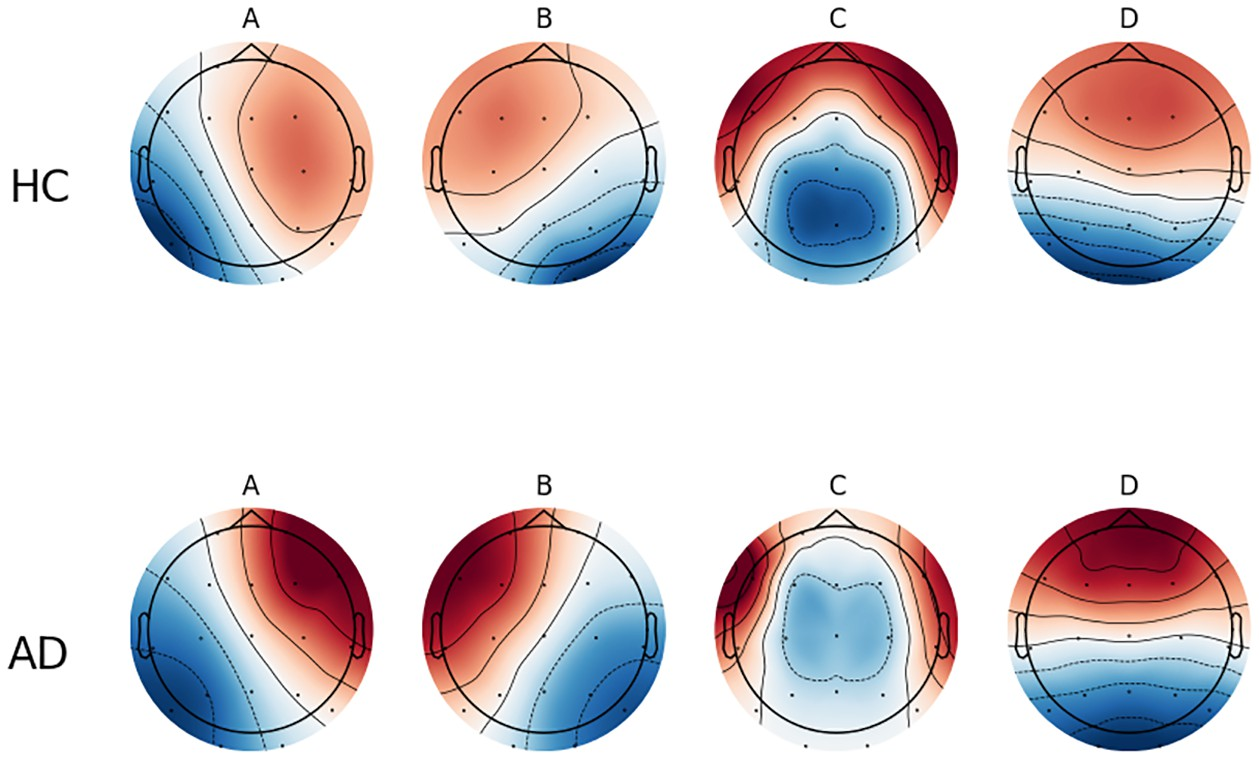
Clustering results of four classical microstates with modified K-mean clustering algorithm.

A considerable number of microstate analysis studies have reported the existence of four prototypical microstate topographies, which are believed to represent brain activity as measured by resting-state EEG. These four topographies include the following: right frontal left posterior, left frontal right frontal occipital midline, and frontal midline, which are types A, B, C, and D, respectively. The results of the clustering analysis of the four microstate topographies for the AD group and the healthy control group are presented in Fig 1. It can be observed that there is a significant difference in microstate C between the two groups. It is important to note that a quasi-steady state of approximately 80–120 ms exists for individual topographies before dynamically transitioning to another EEG topography [33]. Consequently, when an EEG is regarded as a topography of potentials that are continually fluctuating at the time level, the entire recording can be examined through a set of topographies that exhibit dynamic fluctuations at discrete time points. Once the optimal number of microstates (four prototype microstates) has been determined, the subsequent step will be to rank and label them using a modified K-means clustering algorithm and a GEV criterion. One of the parameters of the K-means clustering algorithm is the iteration. This means that the iteration is maintained at each iteration of the K-means algorithm until some stopping criterion is satisfied (a convergence threshold). The algorithm is stopped when the relative error between subsequent iterations changes below the threshold.

**Fig 2:**
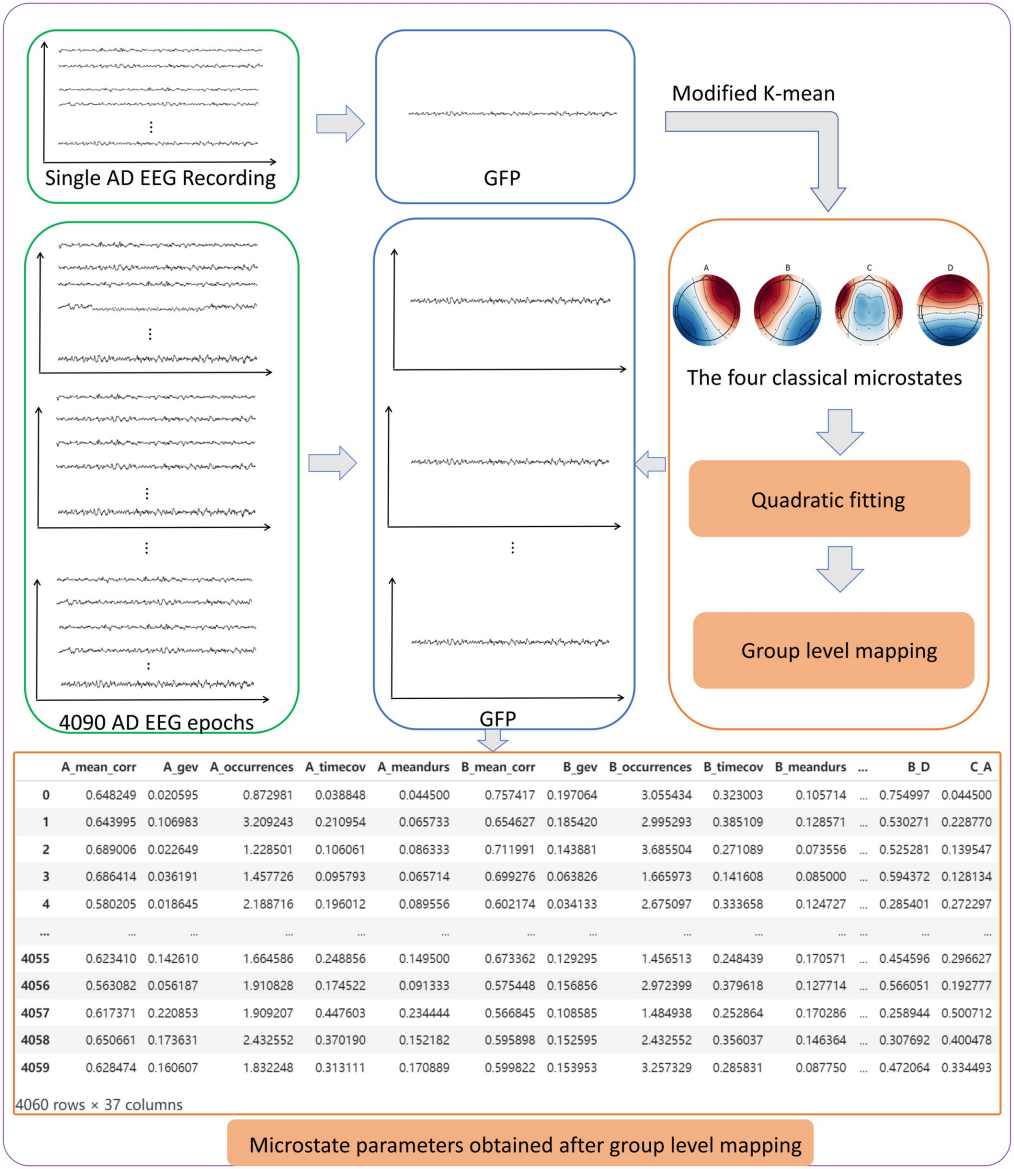
Extraction process of microstate features and conventional features.

**Fig 3:**
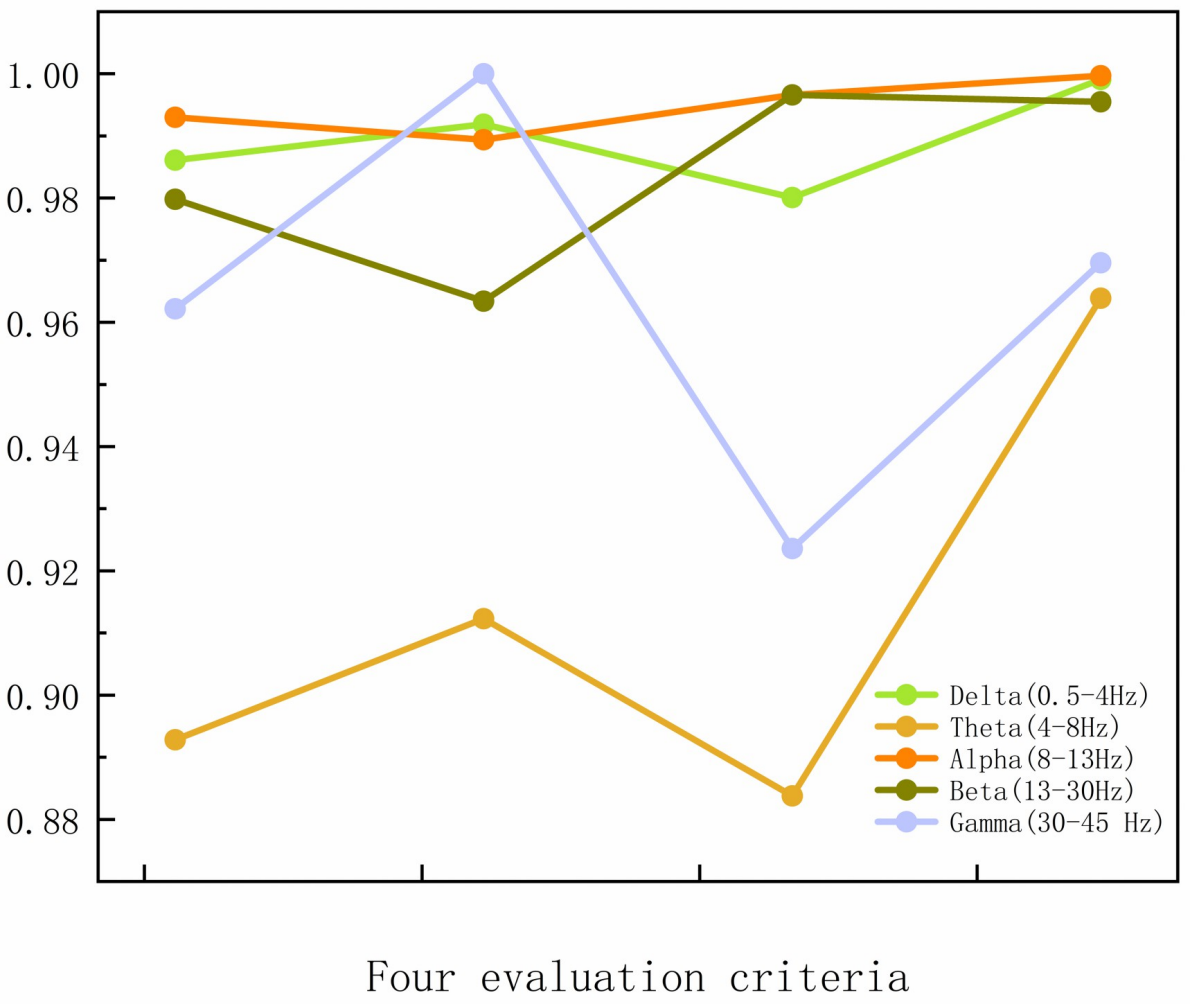
Classification effect of EEG microstate features in different frequency bands in SVM.

The preceding steps are simplified as shown in Fig 2. First, the GFP of a single sample is calculated and the obtained data is subjected to K-mean clustering. Subsequently, the four microstate clustering result sets are horizontally mapped to the GFP of each individual sample. After the algorithm extracts the microstate parameters of the four microstates of each sample and obtains the microstate feature set, the features of this feature set include duration, coverage, and transition probability. As illustrated in Fig 3, the EEG topographies of microstate B and microstate C in AD patients and healthy controls exhibit notable differences. Additionally, previous studies have demonstrated that AD patients exhibit alterations in the temporal features of microstates, including variations in microstate duration. These findings suggest that microstate feature parameters may serve as potential markers for AD classification.

### Conventional EEG feature extraction

In this study, linear and nonlinear features were extracted from EEG microstates in the 8–13 Hz sub-band, which are commonly used for EEG analysis. The linear features include mean, median, root mean square, standard deviation and variance, average power spectral density, spectral flatness, Hjorth parameter, skewness and kurtosis. The nonlinear features extract the Petrosian fractal dimension (PFD), Lempel-Ziv complexity (LZC), and entropy (approximate entropy, sample entropy, and fuzzy entropy).

#### Linear feature extraction

The time-frequency characteristics utilized in the processing of the signal in the time-frequency domain are represented by the equations provided in Table 2.

**Table 2:** Calculation formula linear features.

Features	Formula
Mean	$\bar{x}=\frac{1}{n}\sum_{i=1}^{n}\;x_{i}$
Standard deviation	$s_{x}=\sqrt{\frac{\Sigma {\left(x_{i}-\bar{x}\right)}^{2}}{n-1}}$
Variance	$S^{2}=\frac{\Sigma {\left(X-\bar{X}\right)}^{2}}{n-1}$
Root of Mean Square	$X_{rms}=\sqrt{\frac{\sum_{i=1}^{N}\;X_{i}^{2}}{N}}$
Averaged power spectrum	$P=\int_{0}^{+\infty }\;G\left(\omega \right)d\omega$
Hjorth	**Activity**(***x***(***t***)) = **var**(***x***(***t***)) $\text{Mobility}(x\left(t\right))=\sqrt{\frac{\text{Activity}(x^{\prime }\left(t\right))}{\text{Activity}(x\left(t\right))}}$ $\text{Complexity}(x\left(t\right))=\frac{\text{Mobility}(x^{\prime }\left(t\right)\text{)}}{\text{Mobility}(x\left(t\right))}$
Skewness	* $Skew=\frac{1}{n}\sum_{i=1}^{1}{\left(\frac{x_{i}-\bar{x}}{S}\right)}^{3}$ *
Kurtosis	$Kurt=\frac{1}{n}\sum_{i=1}^{1}{\left(\frac{x_{i}-\bar{x}}{S}\right)}^{4}$

The Hjorth parameter is a method for quantifying the degree of activity and structure of a signal. It was first proposed by Dane Hjorth in 1935 [34]. The concept of measuring and categorizing time-series signals by capturing their dynamics is referred to here. The Hjorth parameter is made up of three main components, namely Activity, Mobility and Complexity. These can be used to indicate the level of activity of a signal, the specific changes in the characteristics and the overall trend of change in order to better represent a given signal.

#### Nonlinear feature extraction

The Petrosian fractal dimension is a fractal dimension employed to characterize the intricacy and self-similarity of a signal or data [35]. It was proposed by Armenak Petrosian in 1995. It is represented by the first row in Table 3. where k denotes the number of signals and N_δ_ denotes the number of variations of the signal.

**Table 3:** Calculation formula nonlinear features.

Features	Formula
The petrosian fractal dimension	**PFD** = **log**_**10**_ ***k***/(**log**_**10**_ ***k*** + **log**_**10**_(***k***/(***k*** + **0.4*****N***_***δ***_))))
Lempel-Ziv complexity	**LZC** = ***c***(***n***)/***b***(***n***)
Approximate Entropy	$\;C_{i}^{m}\left(r\right)=\frac{1}{N-m+1}\text{num}\left\{d\left[X\left(i\right),X\left(j\right)\right]<r\right\}$ $\Phi^{m}\left(r\right)=\frac{1}{N-m+1}\sum_{i=1}^{N-m+1}\;ln\;C_{i}^{m}\left(r\right)$ ***ApEn***(***m***, ***r***, ***N***) = **Φ**^***m***^(***r***) − **Φ**^***m***+**1**^(**r**)
Sample Entropy	$B^{m}=\frac{1}{N-m+1}\sum_{i=1}^{N-m+1}\;\frac{n_{i}^{m}}{N-m+1}$ ***SampEn***(***q***, ***m***, ***N***) = −**log**(***B***^***m***+**1**^/***B***^***m***^)
Fuzzy Entropy	$C_{i}^{m}\left(r\right)=\frac{1}{k}\sum_{j=1,j\neq i}^{k}\;A_{ij}^{m}$ $\varphi^{m}\left(r\right)=\frac{1}{k}\sum_{i=1}^{k}\;C_{i}^{m}\left(r\right)$ ***FuEn***(***m***, ***r***, ***n***) = **ln *φ***^***m***^ (***r***) − **ln *φ***^***m***+**1**^ (***r***)

LZC is a method for characterizing the rate at which new patterns emerge in a time series. This method was initially proposed by Lempel and Ziv [36], hence the name Lempel-Ziv complexity. It is represented by the second row of Table 3, where $\underset{m\to \infty }{lim}\;c\left(n\right)=b\left(n\right)/{\text{log}}_{2}n$.

Approximate Entropy (ApEn) is a nonlinear dynamics parameter utilized to quantify the regularity and unpredictability of fluctuations in a time series [37]. It expresses the complexity of a time series as a non-negative number, reflecting the likelihood of the occurrence of new information in the time series. The greater the complexity of a time series, the greater the ApEn.

Sample entropy is a new time series complexity characterization parameter proposed by Richman et al. in 2000 [38]. Approximate entropy is employed to enhance sample entropy, which gauges the intricacy of time series and the probability of generating novel patterns when dimensionality fluctuates. The greater the probability of generating a new pattern, the higher the sequence’s complexity, and thus, the higher the entropy value.

Fuzzy entropy and sample entropy are analogous in their physical meaning, both of which quantify the probability of a time series generating a new pattern as the dimensionality changes [39]. The probability of a sequence generating new patterns is directly proportional to the degree of complexity of the sequence and inversely proportional to the entropy value.

### Classification

In the context of scientific research, the assessment of performance is a crucial aspect of evaluating the efficacy of each model employed [40]. The objective of this study was to assess the suitability of microstate features for classification with Alzheimer’s disease (AD) and to determine whether they are more effective than conventional electroencephalogram (EEG) features. To this end, several machine learning algorithms were employed to compare the classification performance of the two types of features. Four parameters were applied to evaluate the performance of the classifiers, namely accuracy, sensitivity, specificity, and area under the curve (AUC). The metrics of precision and recall are useful for measuring the efficacy of predictors in classes that are imbalanced [41]. Precision is a measure of the relevance of the results, while recall is a measure of the truly relevant results that are returned.

A true positive (TP) is defined as a model prediction that correctly identifies a sample as belonging to a positive class. Conversely, a false positive (FP) is a model prediction that incorrectly identifies a sample as belonging to a positive class. A false negative (FN) is a model prediction that incorrectly identifies a sample as belonging to a negative class. Finally, a true negative (TN) is a model prediction that correctly identifies a sample as belonging to a negative class. Subsequently, the accuracy, recall, and specificity were calculated based on the TP, FP, FN, and TN values.

The receiver operating characteristic (ROC) curve is the curve with recall on the y-axis and specificity on the x-axis. The area under the ROC curve (AUC) is a metric used to assess the performance of a model. In practical terms, an AUC of greater than 70% is a desirable outcome [42].

The selection of classifiers was based on the stability and simplicity of various known classifiers from previous EEG studies and other biomedical engineering research, including support vector machine (SVM), logistic regression (LR), random forest (RF), and k-nearest neighbor (KNN). To compare the classification performance of microstate EEG features and conventional EEG features, all classifiers were applied to the combined features, resulting in an accuracy rate.

## Results

### Definite major frequency band

Prior to undertaking microstate analysis, it is necessary to identify the EEG band that contains the most characteristic information about AD patients. This process involves the removal of low-correlation and irrelevant EEG bands, thereby further purifying the raw EEG. It also provides more accurate feature information, which in turn leads to more accurate classification results. The aforementioned process was subjected to an analysis utilizing SVM (Gaussian kernel). Subsequent to the testing of the samples, it was determined that the Alpha (8–13 Hz) band exhibited superior accuracy, sensitivity, and specificity in comparison to the other bands as illustrated in Table 4. This is consistent with the performance of previous studies [43]. Therefore, Alpha-band filtering is used in the microstate feature and conventional feature extraction process to obtain the final feature set.

**Table 4:** Test results for each sub-band.

Sub-Band	Accuracy(%)	Sensitivity(%)	Specifitity (%)
Delta(0.5-4Hz)	98.44	99.49	97.43
Theta(4-8Hz)	97.54	99.83	95.34
Alpha (8-13Hz)	99.75	99.66	99.84
Beta(13-30Hz)	97.12	95.12	99.03
Gamma(30–45 Hz)	93.51	100	87.32

### Comparison of microstate features and conventional feature classification

**Fig 4:**
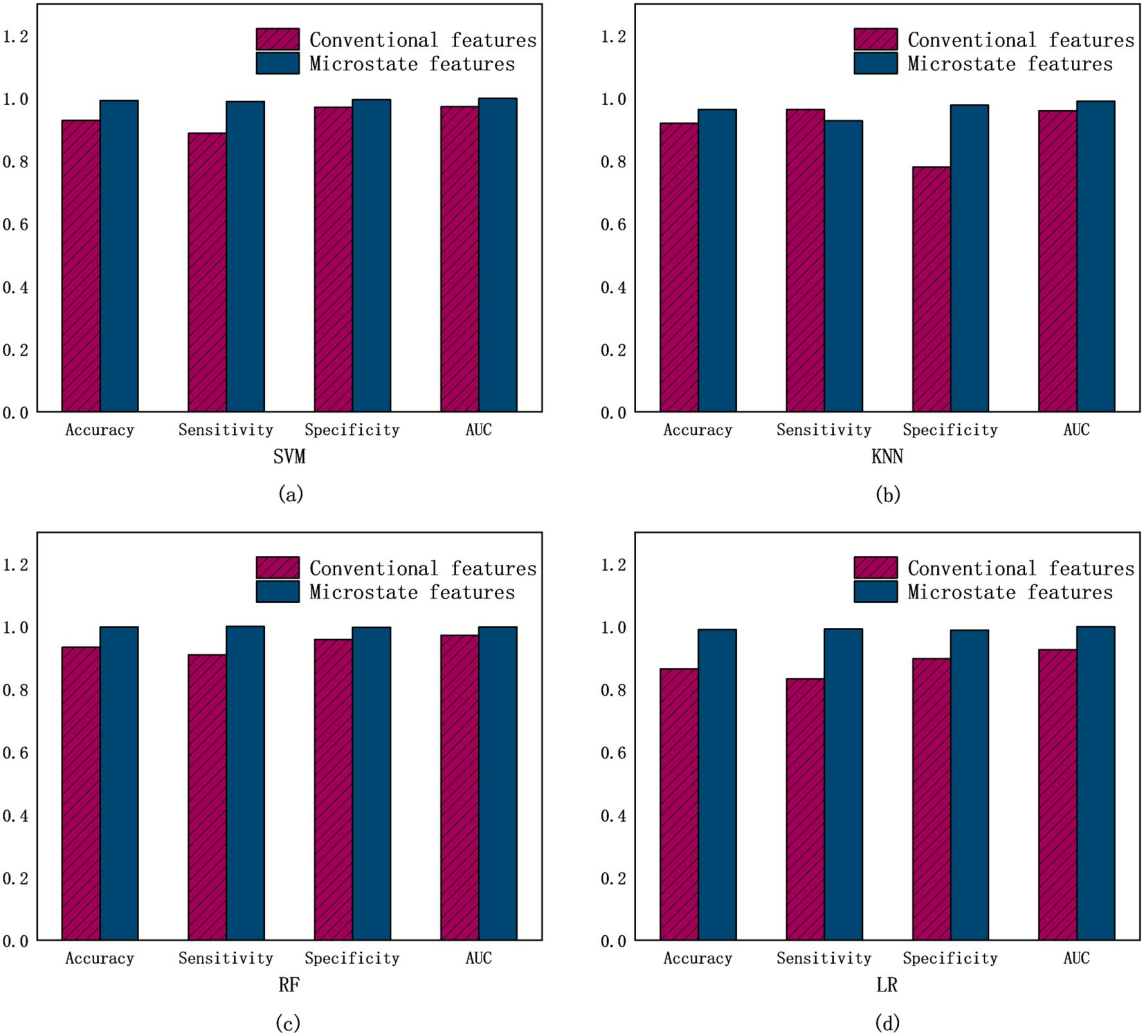
The bar chart represents the values of the four evaluation criteria obtained by feeding the four classifiers into the microstate feature test set and the regular feature test set respectively.

In order to verify that microstate features outperform regular EEG features in the classification performance of AD, four different machine learning classifiers were employed: support vector machine, KNN, random forest, and logistic regression. The classification performance of the four classifiers for the two feature sets is presented in Table 5. These evaluation criteria are visually represented in a bar chart depicted in Fig 4. The results demonstrated that the microstate feature set outperforms the regular EEG feature set in all four of these classifiers, which can be shown to be independent of the classifier selection. Consequently, after accounting for the influence of classifiers, it can be demonstrated that microstate features are more effective than conventional EEG features in the classification of Alzheimer’s disease.

**Table 5:** Classification results of four classifiers for two feature sets.

Classifier	Feature set	Accuracy(%)	Sensitivity(%)	Sensitivity(%)	AUC (%)
SVM	Microstates	99.22	98.94	99.50	99.92
	Conventional	92.89	88.79	97.09	97.23
KNN	Microstates	96.35	92.77	97.75	99.00
	Conventional	91.99	96.33	78.00	96.00
RF	Microstates	99.88	100	99.75	99.87
	Conventional	93.39	90.98	95.85	97.21
LR	Microstates	99.01	99.18	98.83	99.96
	Conventional	86.49	83.34	89.71	92.62

## Discussion

In this study, microstate analysis of resting-state EEG recordings yielded effective features that could successfully distinguish patients with Alzheimer’s disease from healthy controls. Furthermore, it was demonstrated that microstate features are superior to conventional EEG feature sets for machine learning classification of AD. Four commonly used machine learning models were employed for testing, and the results were found to be independent of the choice of classifier. The microstates of several EEG signals from AD patients and healthy controls were analyzed across different frequency bands. Microstate parameters and microstate sequence feature sets were extracted based on state analysis. These were inputted into an SVM classifier to classify the EEG signals from AD patients. The most suitable band for classification was found to be the alpha band. All subjects’ EEG signals were initially filtered in the alpha band, and then the subsequent feature extraction was performed. The microstate parameters extracted from the microstate sequences include duration, number of occurrences, coverage, transition probability, and average correlation coefficient. Conventional EEG signal features included mean, median, root mean square, standard deviation, and variance; mean power spectral density; spectral flatness; Hjorth parameter; skewness; kurtosis; Petrosian fractal dimension; Lempel-Ziv complexity and entropy (approximate entropy, sample entropy, and fuzzy entropy).

In 2021, Lian et al. conducted a study in which they analyzed artificially corrected resting-state EEG arousal microstates in 43 subjects in each of two groups: those with Alzheimer’s disease (AD) and those without the disease (healthy controls). The researchers found that there were overall differences in the duration, incidence, and coverage of the microstates between the two groups. Significantly more duration and coverage were found in the AD group compared to the healthy control group, and increased transitions from microstate A to microstate B were found only in the AD group. Moreover, the probability of transition from microstate A to microstate B showed a negative correlation with the scores of the Brief Mental State Examination (MMSE) scale [44]. In accordance with the grouping and MMSE scores presented in Table 1. As Lian et al. did not attempt to classify AD based on microstate features, the advantage of microstate parameters was not validated by Lian et al. In 2023, Alexandra et al. employed a machine learning framework to investigate time-domain features in AD patients in comparison to age-matched healthy controls [45]. Over 150 time-domain features were extracted from EEG data, including local and distributed evoked activity-related features. These features were then fed into a random forest classifier, which yielded a classification accuracy of 92.95% and a specificity of only 87.94%. It has been shown [46] that when looking at microstates in specific frequency bands, it is found that microstate A is affected in 1–4 Hz, 4–8 Hz, and 13–30 Hz, while microstate D is affected only in the 1–4 Hz and 4–8 Hz bands. The accuracy of the microstate feature classification model in distinguishing between the healthy control and AD groups was 69.8%. In this study, we selected two classification models for comparison: the microstate feature parameter classification model and the conventional EEG feature classification model. Furthermore, four distinct machine-learning classifiers were selected to ascertain the superiority of the microstate parameter classification model over the conventional EEG feature classification model in terms of performance. The EEG microstate parameters were employed as a classification model to differentiate between the resting EEG of AD patients and the resting EEG of healthy subjects, following the application of a machine learning-based SVM classifier. The classification accuracy was found to be 99.2% in the 8–13 Hz band for both the AD and healthy groups. Upon rooting the four classical EEG topographies, it was determined that microstate B and microstate C are most affected in the 8–13 Hz frequency band. The difference produced by this effect is also the reason why the microstate features can recognize AD versus healthy controls.

EEG provides real-time readout of neural functions and network capabilities in different brain states on both temporal and spatial scales, which cannot be achieved by other methods [47]. EEG signals exhibit both linear and nonlinear characteristics. A variety of models have been developed for different EEG signals, and feature extraction and feature classification are widely used in the diagnosis of various diseases. In this study, we also combined the high temporal resolution of the EEG microstates, extracted the EEG signal features at different frequencies of the EEG microstates time series, and processed the microstates feature classification model using an SVM classifier. Our findings revealed that in the frequency band of 8–13 Hz, the accuracy of the classification was 99.3%. In this study, the frequency band of 8–13 Hz was utilized for processing as a whole, and the classification accuracies of all four of our machine learning classification models were found to be relatively high. Additionally, the conventional EEG feature model was input into the four classifiers, and the results obtained were compared with those of the microstate parameter classification model. In contrast, the microstate parameter model was successfully employed to distinguish the AD group from the healthy control group. Furthermore, the machine learning classification effect of the microstate parameter model was demonstrated to be superior to that of the conventional EEG classification model. This study is not without limitations. Due to the paucity of publicly available EEG datasets on AD, the study was constrained to a limited number of samples. Furthermore, the study identified AD as a whole, rather than specifically classifying its various stages. Despite these limitations, we conducted this study for validation purposes and look forward to contributing to the results of this study in the future.

## Conclusions

The objective of this study was to investigate the identification of AD using microstate parametric models and to validate the performance of these models in comparison to conventional EEG models. Two classification models were extracted by employing conventional feature extraction techniques in conjunction with microstate analysis. Four machine learning classifiers were then utilized for classification. The results indicated that the majority of the information pertinent to AD microstate analysis was concentrated within the alpha (8–13 Hz) band. Furthermore, the accuracy of the microstate classification model for this band was found to be 99.2%. The results of four different classifiers (SVM, KNN, RF, and LR) exclude the effect of classifiers and successfully verify that the classification performance of the microstate classification model group is higher than that of the conventional EEG feature classification model group. While the classification results did improve, it is evident that what AD patients truly require is a higher level of recognition efficiency. In this study, we conducted a thorough examination of the data and identified several areas that require further attention. The raw data for this study was subjected to preprocessing in order to render it suitable for analysis. Following the preprocessing stage, the raw data must be subjected to further scrutiny to identify any additional features that may be pertinent to the study. It is anticipated that subsequent work will introduce the use of deep learning. This will permit direct classification following data preprocessing, thereby enhancing the efficiency of AD patient identification. Furthermore, it will ensure that AD patients can receive timely treatment, thereby delaying the course of the disease.

## Supporting information

S1 Table 16 conventional EEG features were obtained from Alzheimer’s disease patients and healthy (control) subjects. (CSV)

S2 Table 36 microstate features of Alzheimer’s patients and healthy (control) subjects were obtained. (CSV)
